# Outcomes of Liver Transplantation with Incidental Intrahepatic Cholangiocarcinoma—Own Experience and a Systematic Review

**DOI:** 10.3390/jcm13154303

**Published:** 2024-07-23

**Authors:** Piotr Remiszewski, Paweł Topolewski, Dariusz Łaski, Anna Drobińska

**Affiliations:** Department of General, Endocrine and Transplant Surgery, Medical University of Gdansk, 80-210 Gdansk, Poland; ptopolewski@gumed.edu.pl (P.T.); dariusz.laski@gumed.edu.pl (D.Ł.); annad@gumed.edu.pl (A.D.)

**Keywords:** liver transplantation, cholangiocarcinoma, incidental cholangiocarcinoma, intrahepatic cholangiocarcinoma, transplant oncology

## Abstract

**Background**: Cholangiocarcinoma, the second most common primary liver cancer, is still a contraindication for performing liver transplantation in most patients. Despite various trials being performed in large clinical centers, the results are still not satisfactory. The aim of this study was to present cases from our own cohort and perform a systematic review of the results of liver transplantation in patients with incidental intrahepatic cholangiocarcinoma. **Materials and methods**: We retrospectively reviewed the records of all patients who underwent liver transplantation and identified two patients with incidental intrahepatic cholangiocarcinoma via histopathological examination of the explanted liver. The results of radiological and biochemical screening performed during liver transplantation, standardized histopathological examination and follow-up data are presented. Additionally, a systematic review of PubMed and Cochrane Reviews based on the PRISMA protocol was performed, yielding 413 similar cases. **Results**: We present two cases of incidental intrahepatic cholangiocarcinoma found after liver transplantation. The patients were managed according to a standard protocol with no consecutive modification of immunosuppression or chemotherapy. There was no recurrence or mortality. In this systematic review, the mean reported number of lesions ranged between 1 and 2 per patient. A total of 42 recurrences were reported. The percentage of recurrences ranged between 28.6% and 80%. **Conclusions**: Despite not being a frequent finding, follow-up and further treatment of patients with incidental iCCA should be reported and analyzed. Extra carefulness in screening is advised in patients who are already diagnosed with oncological disease of the liver. In long-term follow-up, recurrence of the disease is rather probable.

## 1. Introduction

Cholangiocarcinoma (CCA), the second most common primary liver cancer with a median survival of 12–24 months [1], is often associated with few treatment options due to disease advancement and localization. CCA may be classified into intrahepatic (iCCA), perihilar or distal CCA. In recent years, several studies have reported that liver transplantation (LT) is an option for improving survival in patients with iCCA [2,3,4]. Incidental findings of previously undetected CCA tumors in explanted livers usually pose a clinical problem in terms of recurrence and overall patient management. The prevalence of incidental iCCA in LT patients is still unknown. The radiological detection and diagnosis of small tumors less than two centimeters in size are challenging, particularly in cirrhotic livers. Recent studies have shown similar survival rates in selected patients with iCCA to those with hepatocellular carcinoma, suggesting that LT may be a treatment option for selected patients with iCCA [5]. The analysis of patients who were transplanted with incidental iCCA is crucial for understanding the natural course of disease and may provide additional evidence regarding the selection of patients.

In this article, we aim to present and evaluate the means of better screening for liver tumors in the LT qualification process and early outcomes of LT in recipients with incidental CCA in the explanted liver based on the experience of an oncological and transplantation center in northern Poland and a systematic review of the literature.

The aim of this study was to evaluate LT outcomes for incidental iCCA confirmed by histopathological examination of the explanted liver, with no prior suspicion of the tumor, in our center and to perform a systematic review on the same subject. We aimed to examine the epidemiology of incidental iCCA in LT centers and treatment outcomes. We also aimed to provide additional evidence for the natural course of the disease in patients who underwent transplantation for misdiagnosed tumors, especially in the context of LT, as a novel option for iCCA treatment in selected patients.

## 2. Materials and Methods

### 2.1. Study Design and Search Strategy

This systematic review was performed according to the PRISMA (Preferred Reporting Items for Systematic Review and Meta-Analyses) protocol. The PubMed and Web of Science databases were searched for relevant articles published in English. The study was conducted in accordance with the Declaration of Helsinki and approved by the Independent Bioethics Committee for Scientific Research at the Medical University of Gdańsk (nr NKBBN/423/2020–2021). The authors state that the systematic review was not registered, and the PRISMA checklist can be found in Appendix A.

### 2.2. Evidence Acquisition

On 13 July 2024, two independent authors searched PubMed and Cochrane Reviews. We used the following keywords: liver transplantation, incidental, and cholangiocarcinoma. The search query was as follows: “Cholangiocarcinoma” [Mesh] AND (transp*) AND (incident*). We used no additional filters or limitations for our search. The initial search returned 62 results. We screened the titles and abstracts of the articles that met the predefined criteria. If the title or abstract provided sufficient evidence for the potential to meet the inclusion criteria (reporting outcomes of liver transplantation in patients with incidental iCCA in explant examination), the article was chosen for full-text analysis. We included all types of studies for which the full text in English was available. After screening, 24 studies were chosen for full-text analysis, 15 of which met predefined inclusion criteria and were analyzed. The following information was extracted from the original papers: first author, year of publication, study design, time of the study, number of patients, number of HCV- and HBV-positive patients, mean number of lesions, mean size of the lesion, number of tumor recurrences and number of suspected malignancies prior to LT, time from transplantation to recurrence and time from recurrence to death. If some of the information was not reported, it was recorded as NR (not reported). All the data extracted from the included studies and used for analysis are available in Table 1 in the manuscript. The authors state that the study was not registered in a public database.

### 2.3. Inclusion and Exclusion Criteria

The authors (P.R., P.T., D.Ł.) discussed conflicts regarding the inclusion of studies and resolved them by consensus. The inclusion and exclusion criteria were defined before the beginning of the search. We included articles reporting incidental findings of CCA in the explanted liver with no prior knowledge of the tumor. Articles were excluded if the presence of iCCA was known before LT, if the diagnosed tumors were not pure CCA (e.g., mixed iCCA/hepatocellular carcinoma with no iCCA group) or if the diagnosed cancer was not iCCA. If multiple publications from the same cohort were available, only the most recent publication was selected. Studies published only as abstracts, posters or reports from meetings were excluded from the analysis. The full-text articles were assessed, and the relevant data were extracted by text reading and manual screening. We searched for studies that met predefined eligibility criteria. The excluded studies can be found in Appendix B.

### 2.4. Evidence Synthesis and Quality Assessment

Table 1. A total of 15 studies met the inclusion criteria. The quality of the included cohort studies was assessed by two independent researchers (P.T. and D.Ł.) using the Newcastle–Ottawa Scale (NOS). The studies were assessed for selection (up to four stars), comparability (up to two stars), and exposure (up to four stars).

## 3. Results

### 3.1. Case Series

We retrospectively reviewed the records of all 227 patients who underwent LT between April 2018 and February 2024 and identified two incidental iCCAs via histopathological examination of the explanted liver. All the data were obtained by accessing the electronic medical records of the patients. All subjects consented to inclusion in the study.

Patients were eligible for LT due to symptoms of end-stage liver cirrhosis. During the qualification process, the patients underwent full oncological screening, including endoscopy, computed tomography examination a few weeks before LT, oncological marker level examination and specialistic evaluation. In none of the patients was the presence of malignancy suspected during qualification for LT. The results of radiological and biochemical screening for LT, standardized histopathological examination and follow-up data are presented in Table 2 and Table 3. The diagnosis of iCCA was made upon examination of the explanted recipient’s liver performed by a pathology specialist. The patients were followed up for at least two and a half years.

### 3.2. Patient 1

A 45-year-old female qualified for LT due to alcoholic liver cirrhosis. The patient had a latent HBV infection, resistance to treatment ascites, esophageal varices with episodes of bleeding and a history of portal vein thrombosis. The patient underwent deceased donor orthotopic LT. On histopathological examination of the explanted liver, a three cm G2 iCCA was found. No postoperative complications occurred. At the three-year follow-up, neither radiological nor biochemical indications of iCCA recurrence were found.

### 3.3. Patient 2

A 53-year-old male qualified for LT due to alcoholic liver cirrhosis. The patient had encephalopathy and esophageal varices with bleeding episodes. The patient underwent deceased donor orthotopic LT. On histopathological examination of the explanted liver, a 1.3 cm G2 iCCA was found. After several weeks, the patient was readmitted to the surgical department due to elevated transaminase activity and bilirubin levels. After short diagnostics, the anastomotic biliary stricture was found and managed endoscopically. At the two-year follow-up, neither radiological nor biochemical indications of iCCA recurrence were found.

## 4. Systematic Review

**Figure 1:**
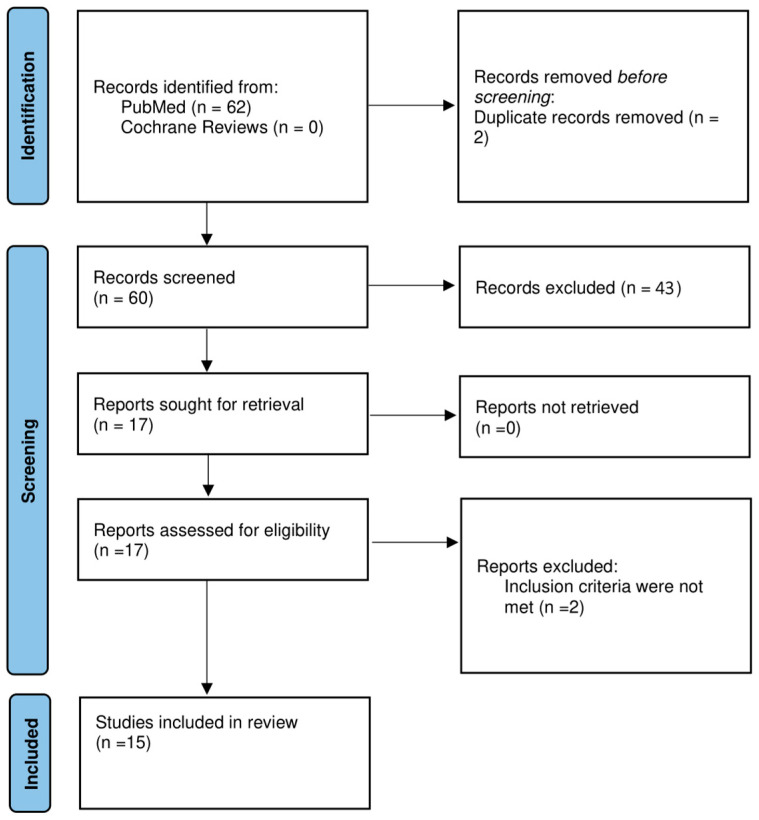
PRISMA protocol for data acquisition.

The study flowchart is presented in Figure 1. Of the 12 articles analyzed, 12 were retrospective, and 3 were case reports. Prospective studies were unavailable due to the nature of clinical issues. The duration of the studies ranged between 7 years and 24 years and 11 months. There was a total of 362 patients, of which 25 were HCV positive and 8 were HBV positive. The mean reported number of lesions ranged between 1 and 2 per patient. The mean reported lesion size ranged between 1 and 4.3 cm per lesion. A total of 42 recurrences were reported. The percentage of recurrences per included study cohort ranged between 28.6% and 80%. There were a total of 35 reported suspected malignancies prior to LT. A summary of the included studies along with the outcomes of LT are presented in Table 1.

## 5. Discussion

The status of LT for iCCA remains an important up-to-date clinical issue with new therapeutic options. Some authors suggest that LT may be an option in some cases involving carefully selected patients [21]. We are clearly not the first to show that even careful screening may result in incidental findings of iCCA in the explanted liver. In our experience, incidental iCCA was found in two explanted livers from 211 LTs. The qualification and transplantation were performed by the same team during the 5 years of the unit’s existence. The iCCA was found in patients who were negative on both radiological and biochemical (Ca 19.9, CEA and AFP) oncological screening. Current screening methods are not definitive, and there are no iCCA-specific exclusion methods except histopathological examination, which is not always possible or necessary. Therefore, cases of incidental iCCA will occur in clinical practice. In the systematic review, suspicion of malignancy varied among the studies; however, in some, in most of the incidental iCCA patients, a high suspicion of malignancy was made. Our experience and systematic review outcomes are consistent regarding the number of lesions and their mean size; in most cases, one to two lesions not exceeding 3 cm were found. Thus, most of the tumors identified through screening are early advanced iCCA. These criteria are features of favorable LT outcomes for iCCA patients [22]. In the short follow-up of our cohort, no iCCA recurrence was observed; however, studies included in the systematic review analysis reported recurrence rates varying from 28.6% to 80%. This difference may be a result of the short follow-up of our patients and differences in the number, size, and biology of the lesions. Therefore, we believe that the quality of evidence regarding the incidence of incidental iCCA and recurrence is low. We believe that the wide range of reported recurrence percentages was influenced by several factors: most “no recurrence” data were based on a small sample and a greater number of suspected malignancies in the explanted liver, and there was major heterogeneity among cohorts in many aspects, such as deceased donor LT and living donor LT, donation type, preliminary liver disease severity, post-LT follow-up and changing protocols of LT qualification, patient management, and follow-up. In most of the studies, the recurrence rate was at least 50%; therefore, we believe that the general assumption of a high chance of tumor recurrence is justified. We believe that in our cohort, the difference may be influenced by the relatively short follow-up period and the early stage at which the tumor was diagnosed in the explanted liver.

In liver transplantation oncological screening, there is little hard evidence regarding cholangiocarcinoma screening. Based on our experience, we suggest that in patients with suspicion of liver cancer, the computed tomography or magnetic resonance imaging should be performed every three months along with biochemical screening. This should minimize the chances of misdiagnosing a liver cancer.

To date, there are no specific follow-up algorithms for the management of incidental iCCA findings. In our center, all patients are managed with a standard protocol (immunosuppression with no further chemotherapy), which may create another clinically important question: should post-transplantation treatment of the incidental CCA cohort vary from standard treatment of the patients? The status of knowledge regarding further management of patients with incidental iCCA is not clear. Routine administration of gemcitabine/5-fluorouracil, doxorubicin and mitomycin to patients with incidental iCCA was reported by Patkowski et al. [23]; however, the recurrence-free survival and long-term results were poor. A change from calcineurin inhibitors to mammalian target of rapamycin inhibitor-based regimens is an option with possible beneficial effects on tumor recurrence and overall survival; however, the number of calcineurin inhibitors is still limited; thus, this therapy is still not a routine option [10,24,25,26]. Most of the authors point to chemotherapy as the main modality of adjuvant treatment if recurrence occurs; however, the type of chemotherapy is still being discussed, and the results are inconsistent. In a review on recurrent iCCA management, the authors suggested that there is also a possibility of surgical treatment (preferably minimally invasive liver resection) and locoregional treatment, such as transarterial chemoembolization, thermal ablation, and stereotactic radiotherapy; however, the data are very limited [27]. The authors also suggested that immunotherapy has potential for the treatment of recurring iCCA; however, we believe that additional evaluation is needed in LT recipients.

In our center, an active watch-and-wait policy was implemented with standard overall management. The patients were managed in an outpatient clinic according to a hepatocellular carcinoma follow-up protocol (routine biochemical and radiological screening and general liver function evaluation every 6 months). We believe that the standard hepatocellular carcinoma protocol is sufficient for the management of our patients; however, recurrences should be reported, as the recurrence rates of iCCA are higher than the recurrence rates of hepatocellular carcinoma, and there is a possibility that the follow-up and screening protocols should be stricter. We found little evidence regarding guidelines for post-LT incidental iCCA screening; therefore, we would like to emphasize the need for reporting the transplant centers’ experiences to create the possibility of future guideline creation. Currently, all patients with iCCA are alive with no indications of iCCA recurrence.

The outcomes of this systematic review indicate the necessity for further studies, as the wide range of outcomes, heterogeneity of cohorts and results do not provide sufficient data for drawing definite conclusions. Conclusions from this systematic review and our case series are that incidental iCCA is more likely to occur in most high-volume LT centers but is rather rare (approximately 1% of all cases). Another general conclusion that can be drawn from a systematic review is that recurrence is rather probable but not definitive. There is also no definite answer to the question of whether incidental iCCA is more likely to be found in patients with suspected malignancy than in patients with no suspected malignancy prior to LT. In our opinion, there is potential for future research regarding the epidemiology of incidental iCCA occurrence in high-volume transplant centers and its future management.

Recent studies have reported that LT is a treatment option for patients with very early iCCA (those whose tumor size is less than two centimeters). These patients can achieve a five-year survival rate of 65% [28]. LT is a better treatment option than resection for patients with iCCA; however, it still yields worse results than the same LT for patients with hepatocellular carcinoma [29]. LT combined with neoadjuvant chemotherapy should be considered a treatment option for patients with locally advanced iCCA [5,30]. Currently, the selection criteria for patients with iCCA undergoing LT are having a very early tumor stage (a single tumor less than two centimeters in diameter) with cirrhosis and having a locally advanced tumor with neoadjuvant chemotherapy. The literature in this field is limited; however, current evidence is a basis for further evaluations of LT as a treatment for iCCA. Incidentally found iCCA also provides information for those studies, as most of the presented studies have relatively long follow-up durations and, therefore, provide reliable information on recurrence rates and survival times. Further studies in this field are warranted.

Novel personalized screening and treatment options could also constitute a new adjuvant therapy option for patients with incidental iCCA. Liquid biopsy is one of the methods that can possibly help in confirming the diagnosis of ambiguous lesions. Serum extracellular vesicles are biomarkers for the early diagnosis and prognostication of CCA [31]. Currently, most targeted therapies are not the first-line therapies for advanced or unresectable iCCA [32]; however, studies have not yet been performed on patients with incidental iCCA after LT. Targeted therapies inhibit specific molecular pathways, potentially resulting in improved treatment responses. The use of targeted therapy for iCCA patients significantly improves patient survival compared with that of patients who receive standard treatment [33]. P53-targeted therapies, MDM2 inhibitors, KRAS-targeted therapies, ATR inhibitors, IDH1 inhibitors, FGFR inhibitors, Her2-targeted therapies, and BRAF and MEK inhibitors are being investigated as targeted therapies for patients with CCA. However, studies have been performed on patients with CCA, and studies on patients after LT have yet to be conducted.

This study proves the importance of careful LT screening, qualification and post-transplantation management for such patients. The main limitations of this study are its retrospective nature (both in our experience and in the studies included in the systematic review), relatively small sample size and lack of data regarding follow-up and screening. Other significant limitations include the significant heterogeneity of the included studies, which makes it difficult to draw conclusions from systematic reviews. The vast time range, heterogeneity of cohorts and included treatments make the included studies difficult to draw specific conclusions from. The limited reporting of treatment protocols is also an additional limitation of this study, as little detailed information on post-transplantation treatment protocols for immunosuppression and their impact on recurrence has been reported. Additionally, the overall quality of the included studies was considered low according to the Newcastle–Ottawa Scale assessment, which affects the reliability of the systematic review outcomes and conclusions. Although the prospective nature of incidental iCCA findings cannot be reached, data regarding screening and follow-up should be reported, as the management of both current and future patients is still an important clinical concern. Future cases of incidental CCA should be reported, and additional data should be analyzed regarding the future management of such patients (ex. specific post-transplant protocol vs standard, future treatment of recurrence). To date, awareness of the general indications and management of LT patients is essential for physicians among transplant teams, especially when it is suitable to refer patients for transplant evaluation.

## 6. Conclusions

Incidental iCCA in the explanted liver is likely a clinical issue that arises in all centers performing LT, as the number of observed incidences of CCA is increasing [34]. The outcomes of our research clearly show that definite guidelines and evidence for the best treatment for patients with recurrent incidental iCCA are lacking. Extra carefulness in screening is advised for patients who are already diagnosed with oncological disease of the liver. During long-term follow-up, recurrence of the disease is probable but not definite. Patients with unclear lesions on screening should be evaluated by a multidisciplinary transplantation team.

## Figures and Tables

**Table 1:** Summary of incidental cholangiocarcinoma histopathological findings.

#	Authors Year Published	Time of the Study	Number of Patients	Mean Number of Lesions	Mean Size of Lesion (cm)	Number (and Percentage) of Tumor Recurrences	Number (and Percentage) of Suspected Malignancies	Recurrence Time after Transplantation	Survival Time after Recurrence
1	Loinaz C et al. [6] 1998	11 years 3 months	2	1.2	1	1 (50%)	NR	NR	58 months
2	Ghali P et al. [7] 2005	7 years	10	NR	NR	10 (100%) Intrahepatic: 4, Extrahepatic: 6, site NR	1 (10.0%)	26 months, median	30 months, median
3	Ali JM et al. [8] 2011	20 years	9	NR	2.05	5 (55.56%) Sites: bone, lungs, small bowels, peritoneal	3 (33.33%)	25.8 months, median	25 months
4	Sapisochin G et al. [9] 2011	10 years	14	1	2.5	8 (57%) Intrahepatic: 1 Extrahepatic: 6, site NR Both: 1	10 (71.43%)	8 moths, median	9 months, median
5	Serra V et al. [10] 2016	15 years	4	NR	3.33	3 (75%) Intrahepatic: 1 Extrahepatic: 2, sites: lungs, bones	4 (100%)	15, 49 months, mean	24, 76 months, mean
6	Takashi K et al. [11] 2016	11 years	13	1.9	2.1	7 (53.8%) Intrahepatic: 1 Extrahepatic: 6, site: lungs, intraperitoneal	5 (38.46%)	13 months, median	NR
7	Elshamy M et al. [12] 2017	10 years 11 months	13	2.0	2.8	1 (30.8%), extrahepatic, site: lungs	11 (84.62%)	35.6 months, median	NR
8	Mitra S et al. [13] 2018	-	1	1	NR	0	0 (0%)	-	-
9	Krasnodębski M et al. [14] 2020	24 years 8 months	8	NR	3.8	6 (75.0%), sites NR	NR	6 months, median	18 months, median
10	Torres MAH et al. [15] 2020	-	1	2	4.3	0	1 (100%)	-	-
11	Ziogas IA et al. [16] 2021	17 years 5 months	286	NR	NR	NR	NR	NR	NR
12	Murta MCB et al. [17] 2022	-	1	1	1.7	0	0 (0%)	-	-
13	Safdar NZ et al. [18] 2022	32 years 7 months	40 (other sites 55)	NR	NR	NR	NR	NR	NR
14	Garcia-Moreno V et al. [19] 2023	13 years 11 months	7	1	NR (largest nodule 2.2 cm)	0	4 (57.14%)	-	-
15	Schwenk L et al. [20] 2023	4	1	3.125	NR	0	NR	-	-

**Table 2:** Summary of histopathological examination results and follow-up data in case series.

#	Age	Sex	Virological Status	Primary Liver Disease	Histopathological Diagnosis	Tumor Size Grade	Follow-Up Length	Recurrence in Radiological Examination	Follow-Up Ca 19.9 (U/mL)	Follow-Up CEA *** (ng/mL)	Follow-Up AFP **** (ng/mL)	Complications
1	45	Female	HBV (+)	ALD *	CCA **	3 cm G2	41 months	No	3.3	2.41	7.29	None
2	52	Male	Negative	ALD *	CCA **	1.3 cm G2	24 months	No	6.0	1.52	2.39	Anastomotic biliary stricture

* Alcohol liver disease, ** Cholangiocarcinoma, *** carcinoembryonic antigen, **** alpha-fetoprotein.

**Table 3:** Summary of case series data.

#	Age	Sex	Virological Status	Time of Last CT * Examination Prior to Liver Transplantation	Ca 19.9 (U/mL)	CEA ** (ng/mL)	AFP *** (ng/mL)	MELD **** Score	Milano Criteria	Up to 7 Criteria	UCSF Criteria
1	45	Female	HBV (+)	5 weeks	7.5	4.72	3.64	11	Qualified	Qualified	Qualified
2	52	Male	Negative	11 weeks	6.5	1.33	2.64	17	Qualified	Qualified	Qualified

* Computed tomography, ** carcinoembryonic antigen, *** alpha-fetoprotein, **** model for end-stage liver disease.

**Figure A1:**
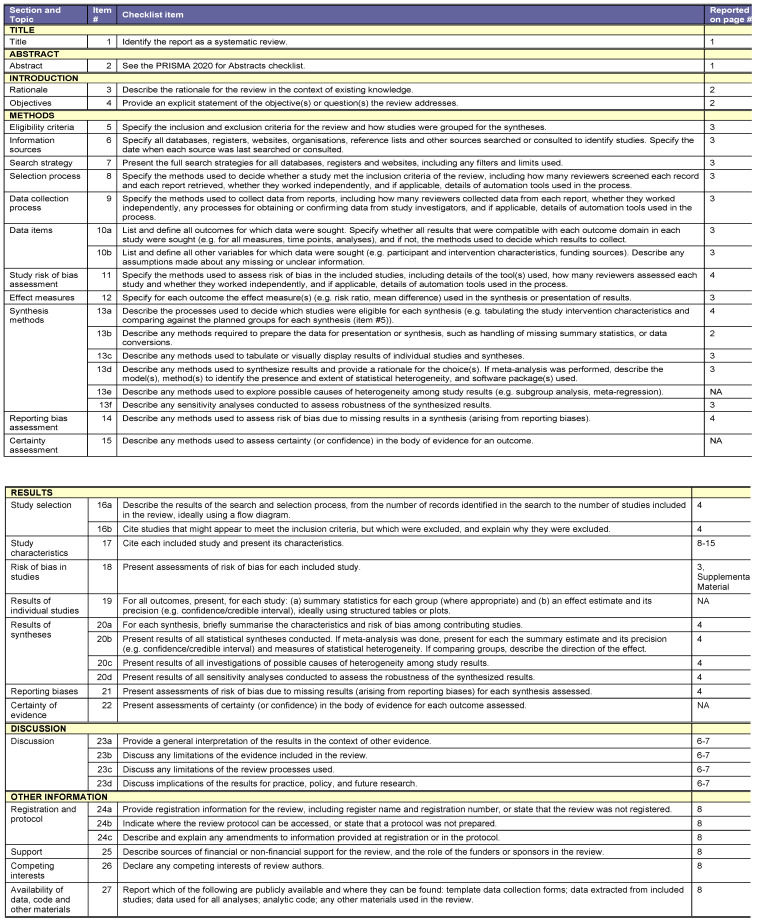
PRISMA checklist [35].

## Data Availability

The data presented in this study are available on request from the corresponding author due to privacy.

## Appendix B

**Table A1:** Reasons for study exclusion.

Authors (Year)	Title	Reasons of Exclusion
Heimback Julie K. (2011) [1]	Dodging the Bullet: Incidental Hilar Cholangiocarcinoma in Patients Undergoing Liver Transplantation	Not compliant with inclusion criteria
Patkowski et al. (2014) [2]	Poor Outcomes After Liver Transplantation in Patients with Incidental Cholangiocarcinoma Irrespective of Tumor Localization	Results duplication
Krasnodębski et al. (2020) [3]	Analysis of Patients with Incidental Perihilar Cholangiocarcinoma: An Old and a Persistent Burden for Liver Transplantation	Results duplication
Hara et al. (2021) [4]	Incidental intrahepatic cholangiocarcinoma in patients undergoing liver transplantation: A multicenter study in Japan	Not compliant with inclusion criteria
